# Different Carbohydrate Ingestion Patterns Do Not Affect Physiological Responses, Whole‐Body Substrate Oxidation or Gastrointestinal Comfort in Cycling

**DOI:** 10.1002/ejsc.12336

**Published:** 2025-06-09

**Authors:** Robyn Owen Jones, Mariana Vaz DE Oliveria, Bethan Palmer, Danny Maguire, George Butler, Isabel Gothard, Kate Kavanagh, Jose Areta, Jamie Pugh, Julien Louis

**Affiliations:** ^1^ Research Institute of Sports and Exercise Sciences (RISES) Liverpool John Moores University Liverpool UK

**Keywords:** endurance exercise, exercise metabolism, performance, sport nutrition

## Abstract

Fuelling during endurance exercise has evolved towards greater amounts of carbohydrates (CHO) ingested per hour, which can prove challenging for athletes. However, the effects of different CHO ingestion patterns during exercise have scarcely been investigated in cycling. 20 recreationally active males cycled for 180 min at lactate threshold on two occasions in a randomised counterbalanced order. Participants consumed 90 g/h of CHO, either as 22.5 g every 15 min or 45 g every 30 min (CHO‐15 and CHO‐30, respectively). Respiratory gases, blood glucose, lactate, heart rate, RPE and gastrointestinal symptoms were assessed every 15 min. Physiological responses showed no difference between conditions or significant interactions, except for blood glucose which saw a greater increase in CHO‐15 during the first 30 min (interaction; *p* = 0.03). Whole body CHO and fat oxidation were not different between conditions (2.38 ± 0.37 and 2.33 ± 0.39 g/min, *p* = 0.25 and 0.19 ± 0.07 vs. 0.22 ± 0.08 g/min, *p* = 0.10 for CHO‐15 and CHO‐30, respectively). Subjective markers of gastrointestinal symptoms did not differ between conditions (*p* > 0.05) except for urge to defecate (*p* = 0.05); however, only 1 participant reported a score > 4 across any symptoms. Ingesting a larger CHO amount at less regular intervals during prolonged cycling had minimal impact on physiological responses to exercise, whole‐body substrate oxidation and gut discomfort, allowing athletes to freely select their preferred strategy.

1

**Summary**

- Different carbohydrate ingestion patterns during 180 min steady state cycling (22.5 g every 15 min or 45g every 30 min) did not affect physiological responses to exercise, whole body substrate oxidation or subjective gut comfort.
- Athletes appear free to choose an appropriate strategy based on personal preference, with minimal impact on whole body exercise metabolism.

## 2 Introduction

The importance of high carbohydrate (CHO) availability during endurance cycling performance (> 90 min) is well established (E. Coyle 1992; Currell and Jeukendrup 2008; Burke et al. 2017). The primary reason being an ability to maintain moderate–high exercise intensities, through a delayed onset of fatigue by withstanding liver glycogen depletion, the maintenance of blood glucose concentration (euglycemia), increased CHO oxidation and stimulating the central nervous system (Gonzalez et al. 2015; Stellingwerff and Cox 2014). As a result, contemporary nutritional guidelines recommend a CHO intake of up to 90 g per hour (g/h) of multiple transportable CHO for prolonged exercise lasting ≥ 2.5 h (Thomas et al. 2016; A. E. Jeukendrup 2004), with recent reports suggesting additional benefits from higher intakes of 120 g/h (Podlogar et al. 2022; Urdampilleta et al. 2020). These practices have allowed exogenous CHO oxidation to reach as high as 1.6 or 1.8 g/min in some studies (Hearris et al. 2022; Jentjens and Jeukendrup 2005), allowing a sparing of limited endogenous liver glycogen stores (Stellingwerff et al. 2007; Gonzalez et al. 2015). However, ingesting such large quantities of CHO can be challenging for endurance cyclists under race conditions (Pfeiffer et al. 2012).

To achieve such high CHO intakes, athletes are required to have regular CHO feedings, typically consuming sports supplements such as energy gels, bars and sports drinks 3–4 times per hour (every 15–20 min). Such strategies can become tedious or impractical (anecdotal evidence from the field), possibly impacting race ‘flow’ and focus, which could prove costly considering races at the elite level are often decided by key moments such as an unpredicted attack or breakaway from the peloton. A less frequent larger CHO bolus could provide a more practical solution. However, maintaining a high CHO intake with less frequent feedings may pose an increased risk of developing gastrointestinal (GI) symptoms (Stocks et al. 2016), with moderate‐severe symptoms likely impairing exercise performance.

Despite decades of work focused on CHO intake during cycling (E. F. Coyle et al. 1986, 1997; Hearris et al. 2022), very little research has focused on different ingestion patterns of the same absolute quantity of CHO. To the authors' knowledge, only one study has previously investigated this in cycling (Fielding et al. 1985), reporting no change in physiological responses or RER during exercise between CHO conditions; however, the quantity of CHO ingested was extremely low considering current contemporary guidelines (21.5 vs. 90 g/h). As CHO availability during exercise is a profound regulator of physiological responses and substrate utilisation (E. F. Coyle et al. 1997; Fell et al. 2021), these data are not applicable to athletes today, who can consume > 4‐fold the CHO quantity per hour compared to the previous study (Saris et al. 1989; Strobel et al. 2022). Several studies have investigated the effects of a glucose bolus pre‐exercise (Krzentowski et al. 1984; Guezennec et al. 1989) and reported similar peak exogenous CHO oxidation rates (0.48–0.65 g/min) when compared to others who investigated more frequent feeding strategies (Massicotte et al. 1989, 1990, 1994). More recently, Stocks et al. (2016) and Mears et al. (2020) specifically investigated the effects of CHO feeding strategies in well trained cross‐country skiers and runners, reporting changes in lipid and exogenous CHO oxidation, respectively, due to manipulated CHO ingestion patterns. However, differences in the feeding strategies and exercise intensities utilised as well as alterations in physiological demands across the sporting disciplines (walking, running and cross‐country skiing) make previous study results not directly transferable to endurance cycling.

As such, there is no cycling‐specific, up to date information available to inform endurance cyclists and practitioners regarding the effects of different CHO ingestion patterns (frequency and dose) during prolonged cycling under conditions of high CHO availability, as all modern‐day endurance cyclists would find themselves during competition. Therefore, the aim of this study was to investigate the effects of different CHO ingestion patterns on physiological responses to exercise, substrate oxidation, GI symptoms and exercise capacity. We hypothesised that there would be no effect of feeding frequencies on physiological responses to exercise, substrate oxidation or exercise capacity. We also hypothesised that there would be moderate reports of GI symptoms across both conditions.

## 3 Methods

### 3.1 Participants

Twenty tier 1 recreationally active males, as outlined by McKay et al. (2022), participated in the study (Table 1). Participants were healthy, training 3–4 times per week and not following CHO‐restrictive diets. Written and verbal information regarding study procedures was provided before written informed consent was obtained. The study was approved by the Liverpool John Moores University Research Ethics Committee. Sample size estimation was determined based on lipid oxidation data of Stocks et al. (2016), where high CHO consumption at a high and low feeding frequency during exercise resulted in mean lipid oxidation values of 0.29 ± 0.12 and 0.24 ± 0.13 g/min. It provided an effect size of 0.4 and corresponded to an a priori sample size of 8 to achieve an alpha of 0.05 and power of 0.95. However, as the low frequency ingestion strategy used by Stocks et al. (2016) was extreme compared to the current study, a more conservative approach was deemed appropriate, using a small–moderate effect size of 0.25 (Cohen 1988) which required an a priori sample size of 18 to achieve an alpha of 0.05 and power of 0.95 (G*Power, version 3.1.9.7).

**TABLE 1:** Participant characteristics.

Participants (*n*)	20
Age (years)	24 ± 3
Body mass (kg)	76.2 ± 7.5
Height (cm)	181.0 ± 5.5
V̇O_2max_ (L/min)	3.82 ± 0.41
V̇O_2max_ (mL/kg/min)	50.4 ± 3.8
PO at LT1 (watts)	139 ± 29
PPO (watts)	314 ± 31
PPO (watts/kg)	4.1 ± 0.4

Abbreviations: PO at LT1, power output at lactate threshold 1; PPO, peak power output; V̇O_2max_, maximal oxygen consumption.

### 3.2 Study Design

**FIGURE 1:**
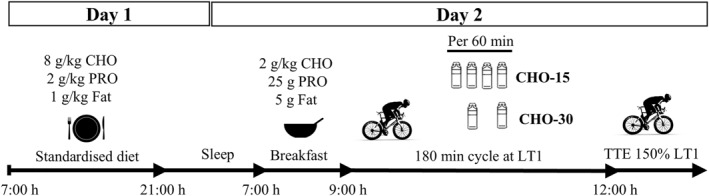
Schematic of study protocol for each condition, where participants followed a standardised high CHO diet for 24 h prior to completing a 180 min steady state cycling protocol followed by an exercise capacity test. CHO, carbohydrates; g/kg, grams per kilogramme of body mass; LT1, lactate threshold 1; PRO, protein; TTE, time to exhaustion.

In a randomised counterbalanced crossover design, participants completed two experimental trials, consisting of 180 min cycling at an intensity equivalent to the first lactate threshold (LT1; defined as baseline blood lactate value +1 mmol/L; Zoladz et al. 1995). Trials were preceded by a 24 h dietary control period, designed to provide high CHO availability. During exercise, participants ingested 90 g/h of CHO in the form of CHO gels, with different ingestion patterns (frequency and dose) of either a 22.5 g CHO gel every 15 min or a 45 g CHO gel every 30 min (Figure 1). All visits were separated by ≥ 7 days.

### 3.3 Preliminary Testing

Height and body mass were measured semi‐nude (SECA, Hamburg, Germany), before participants were seated for resting measures of heart rate (Polar H10, Kempele, Finland), blood glucose and lactate using capillary fingertip blood samples, which were immediately analysed (Biosen C‐Line, EKF Diagnostics, Cardiff, UK). Participants then completed incremental lactate threshold and maximal oxygen consumption (V̇O_2max_) tests on a cycle ergometer (Lode, Groningen, Netherlands). Briefly, participants began cycling at 100 W and exercise intensity increased 25 W following each 4 min stage. In the final 30 s of each stage, a fingertip blood sample was collected and immediately analysed for blood glucose and lactate, with ratings of perceived exertion (RPE; Borg 1982) and heart rate also being collected in the final 10 s of each stage. The test was terminated when participants reached the onset of blood lactate accumulation ≥ 4 mmol/L (Heck et al. 1985).

Following 10 min rest, participants returned to the ergometer to complete a V̇O_2max_ test. The test began at 100 W and exercise intensity increased 25 W every 1 min until volitional exhaustion. Gas exchange was measured continuously throughout using a metabolic cart (Vyntus CPX, Vyaire Medical, Chicago, USA), with V̇O_2max_ defined as the highest V̇O_2_ sustained over a 30 s average. Heart rate and RPE were collected in the final 10 s of each stage and were used as indirect markers of volitional exhaustion. Peak power output (PPO) was determined to describe participant characteristics using the equation outlined by Kuipers et al. (1985). Following a further 10 min of rest, participants began a familiarisation period where the experimental procedures (subsequently described) were replicated identically for 60 min, followed by an exercise capacity test. No CHO was ingested during the familiarisation, with participants provided with 150 mL of water every 15 min.

### 3.4 Pre‐Experimental Controls

Twenty four h prior to both experimental trials, participants were asked to follow a meal plan designed to replicate pre‐competition practices of endurance athletes. In line with contemporary nutritional guidelines, the meal plan provided 8, 2 and 1 g/kg of body mass/day (g/kg/d) of CHO, protein and fat, respectively. The plan provided ∼1 L of fluids, with participants also instructed to consume a further 1–1.5 L of water throughout the day. On the morning of the experimental trial, participants were asked to consume a high CHO breakfast, containing 2 g/kg of body mass (g/kg) of CHO, 25 and 5 g of protein and fat, respectively, as well as ∼500 mL of fluids. Meal plans instructed participants to consume foods and beverages at usual mealtimes in the form of breakfast, lunch and dinner, with various snacks between meals. To make the quantity of food more tolerable, the meal plans provided a healthy mixed diet, supplemented with additional high glycaemic index foods and high CHO beverages. Plans contained fruits, fruit juice, oats, bread, jam, rice, chicken, sweet chilli sauce and small portions of vegetables (to minimise intake of dietary fibre) and were created using an online nutrition software (Nutritics, Dublin, Ireland). To confirm adherence, participants were asked to send timestamped photographs of meals prior to consumption using online messages throughout the control period (WhatsApp, Meta, California, USA). Participants were free to consume water as desired but were asked not to consume any calorie containing beverages or foodstuffs outside what was outlined within the plan. Additionally, no exercise, consumption of caffeine or alcohol was permitted 24 h prior to each experimental trial.

### 3.5 Experimental Trials

On the morning of each trial (9:00 AM ± 40 min; standardised within participant), at least 60 min post‐breakfast consumption (between 6 and 8 a.m., with time replicated across conditions), participants were seated for collection of resting values of heart rate, blood glucose and lactate, completed a standardised cycling warm up (10 min at 100 W) and then cycled for 180 min at an intensity corresponding to LT1 (139 ± 29 W). In the final 30 s of every 15 min, capillary blood samples were collected for determination of blood glucose and lactate, with heart rate and RPE also collected. Expired gas was collected and averaged for the final 2 of every 15 min. Following this measurement, subjective GI symptoms were recorded on a 0–10 scale (nausea, reflux, stomach fullness, abdominal cramps, flatulence and urge to defecate) with scores of 0, 5 and 10 indicating no discomfort at all, moderate and unbearable discomfort, respectively (Wilson 2017).

Dependent on experimental condition, participants were provided with 6 or 12 CHO gels in total, consuming either 22.5 g CHO every 15 min or 45 g CHO every 30 min (CHO‐15 and CHO‐30, respectively). These specific CHO doses were based on anecdotal evidence from the field, typical feeding strategies used at the elite level and CHO doses of commercially available gels (e.g., Science in sport CHO gels; 22 g or 40 g CHO). Gels were specifically formulated for the purpose of the study with a combination of glucose and fructose at a 1:0.8 ratio (Keto Life, Lancashire, UK), in line with CHO intake recommendations during exercise lasting > 2.5 h (Thomas et al. 2016). 150 mL of water was also provided every 15 min to maintain and standardise hydration status. Every 30 min following the consumption of a gel and prior to the ingestion of water, participants were asked to provide a 0–10 score for the perception of sweetness and the desire to consume CHO, where 0, 5 and 10 indicated no sweetness, perfectly sweet and unbearably sweet or no desire, moderate desire and strong desire, respectively.

Following the 180 min, once all measures were collected and the final 150 mL of water was consumed, participants began a time to exhaustion cycling test at an intensity corresponding to 150% LT1 (209 ± 43 W). Participants cycled until volitional exhaustion, defined as an inability to maintain a cadence > 60 rpm for 10 s consecutively. Participants were allowed to consume water *ad libitum* during this period. Participants were not directly told their performance time; however, total exercise time, cadence and fixed power output were visible.

Respiratory gases were used to determine mean whole‐body CHO and fat oxidation (g/min) for every 15 min of the 180 min cycle at LT1 using stoichiometric equations (A. E. Jeukendrup and Wallis 2005). Total exercise energy expenditure was estimated assuming 1 g of CHO and fat corresponded to 17.57 and 39.33 kJ, respectively (Ferrannini 1988).

### 3.6 Statistical Analysis

All statistical analyses were conducted using SPSS version 29 (IBM, Chicago, United States). All data were checked for normality using the Shapiro–Wilk test. Two‐way repeated measures ANOVA were used to determine interactions and main effects for condition and time for physiological responses to exercise (heart rate, RPE, V̇O_2_, blood glucose and lactate) and substrate oxidation (RER, CHO and fat oxidation (g/min), exercise energy expenditure and % contribution of CHO and fat to exercise energy expenditure). Significant main effects were further analysed using Bonferroni post hoc tests to explore where significant differences occurred. ANOVA effect sizes are partial eta^2^ (*η* ^2^_*p*_) with values of 0.01, 0.06 and 0.14 corresponding to a small, medium and large effect, respectively (Cohen 1988). Total CHO and fat oxidation (g) and exercise capacity were analysed using the Wilcoxon ranked test (nonparametric equivalent to paired *t*‐test) as data were not normally distributed. Cohen's *d* effect sizes were calculated by dividing the standardised test statistic (Z score) by the square root of the number of observations, with an effect size of 0.2, 0.5 and 0.8 equating to a small, moderate and large effect, respectively (Cohen 1988). One participant was excluded from the analysis for stomach fullness as they reported a severe score of 7 throughout exercise for both conditions, which based on his physical appearance, suggested lack of understanding of the severity of symptom scores. All values are presented as means ± SD unless otherwise stated, with significance set at *p* < 0.05.

## 4 Results

### 4.1 Physiological Responses

Heart rate, RPE and absolute V̇O_2_ responded similarly in both trials, with no main effect for condition (*p* = 0.22; *p* = 0.46 and *p* = 0.82, respectively) and no significant interaction (*p* = 0.37; *p* = 0.49 and *p* = 0.32, respectively). Heart rate, RPE and absolute V̇O_2_ increased throughout exercise time (*p* < 0.001, for all variables), reaching significance versus the first time point following 15, 30 and 150 min, respectively (Table 2).

**TABLE 2:** Heart rate, rating of perceived exertion (RPE), oxygen uptake (V̇O_2_) and total energy expenditure (TEE) during 180 min steady state cycle with high CHO availability consuming either 22.5 g every 15 min (CHO‐15) or 45 g every 30 min (CHO‐30).

Condition	Exercise time (min)	Heart rate (beats/min)	RPE	V̇O_2_ (L/min)	TEE (kJ/min)
CHO‐15	15	132 ± 14	10 ± 2	2.15 ± 0.48	46.2 ± 10.0
	30	132 ± 13	10 ± 2^a^	2.23 ± 0.47	48.0 ± 10.1
	45	133 ± 15	10 ± 2^a^	2.31 ± 0.39	49.8 ± 8.0
	60	135 ± 14	11 ± 1^a^	2.32 ± 0.40	50.1 ± 7.9
	75	136 ± 14	11 ± 2^a^	2.35 ± 0.40	50.2 ± 8.2
	90	136 ± 14	12 ± 2^a^	2.35 ± 0.42	50.4 ± 8.5
	105	136 ± 14	12 ± 2^a^	2.38 ± 0.42	51.0 ± 8.5
	120	138 ± 14^a^	12 ± 2^a^	2.37 ± 0.42	50.5 ± 8.6
	135	140 ± 17^a^	12 ± 2^a^	2.39 ± 0.44	51.3 ± 8.5
	150	140 ± 16^a^	13 ± 2^a^	2.40 ± 0.44^a^	51.4 ± 9.0
	165	142 ± 13^a^	13 ± 2^a^	2.41 ± 0.42^a^	51.4 ± 8.5
	180	144 ± 16^a^	13 ± 2^a^	2.44 ± 0.40^a^	51.8 ± 7.9
CHO‐30	15	132 ± 12	10 ± 2	2.20 ± 0.39	47.1 ± 8.4
	30	131 ± 14	10 ± 2^a^	2.28 ± 0.42	49.1 ± 8.7
	45	137 ± 14	11 ± 2^a^	2.30 ± 0.42	49.4 ± 8.4
	60	136 ± 13	11 ± 1^a^	2.32 ± 0.41	50.3 ± 8.0
	75	136 ± 14	11 ± 2^a^	2.34 ± 0.43	50.1 ± 8.7
	90	137 ± 15	11 ± 2^a^	2.34 ± 0.41	50.3 ± 8.1
	105	139 ± 14	11 ± 1^a^	2.35 ± 0.44	50.4 ± 8.4
	120	140 ± 16^a^	12 ± 2^a^	2.38 ± 0.42	50.9 ± 8.4
	135	143 ± 15^a^	12 ± 2^a^	2.38 ± 0.43	50.6 ± 8.3
	150	144 ± 14^a^	12 ± 2^a^	2.41 ± 0.43^a^	51.3 ± 8.6
	165	144 ± 15^a^	13 ± 2^a^	2.41 ± 0.40^a^	51.3 ± 7.8
	180	147 ± 16^a^	13 ± 2^a^	2.44 ± 0.41^a^	52.0 ± 7.8

^a^ Significant difference versus 15 min time point.

**FIGURE 2:**
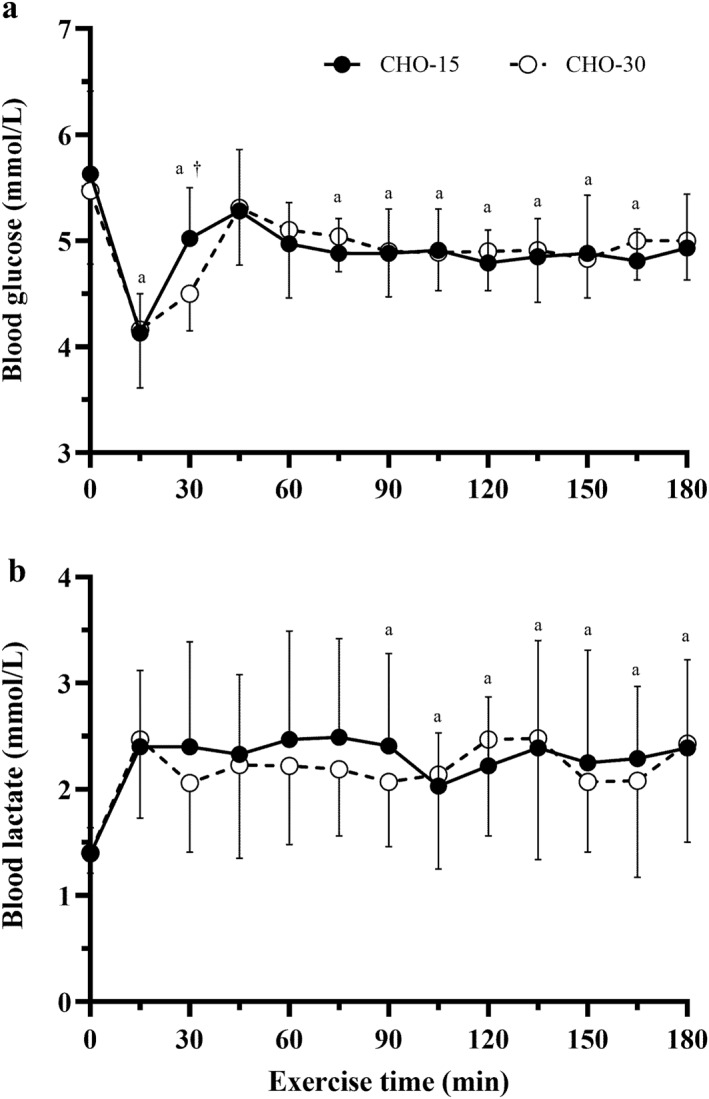
Plasma glucose (a) and lactate (b) concentration during 180 min steady state cycling with high CHO availability through different CHO ingestion patterns of 22.5 g CHO every 15 min (CHO‐15) or 45 g CHO every 30 min (CHO‐30). Data presented as means ± SD. ^a^ Significantly different from first timepoint. ^†^Significant interaction, where conditions responded differently at the 30 min time point.

Blood glucose and lactate were similar in both CHO‐15 and CHO‐30 (*p* = 0.96, *η* ^2^_*p*_ < 0.001; *p* = 0.34, *η* ^2^_*p*_ = 0.05, respectively). Blood glucose decreased from rest following 15 min of exercise in both conditions (main effect for time: *p* < 0.001 and *η* ^2^_*p*_ = 0.56), before concentrations increased at 30 min. This increase was greater in CHO‐15 versus CHO‐30 (+0.93 and 0.34 mmol/L, respectively; interaction effect: *p* = 0.03, *η* ^2^_*p*_ = 0.11). At 45 min of exercise, glucose concentrations were again comparable, which was maintained thereafter (Figure 2a). Blood lactate saw no significant interaction (*p* = 0.42); however, there was a significant main effect for time (*p* < 0.001 and *η* ^2^_*p*_ = 0.27) as lactate increased similarly from rest (+42.2 and +43.1% for CHO‐15 and CHO‐30, respectively) and remained elevated in both conditions throughout exercise (Figure 2b; *p* < 0.01).

### 4.2 Substrate Utilisation

**FIGURE 3:**
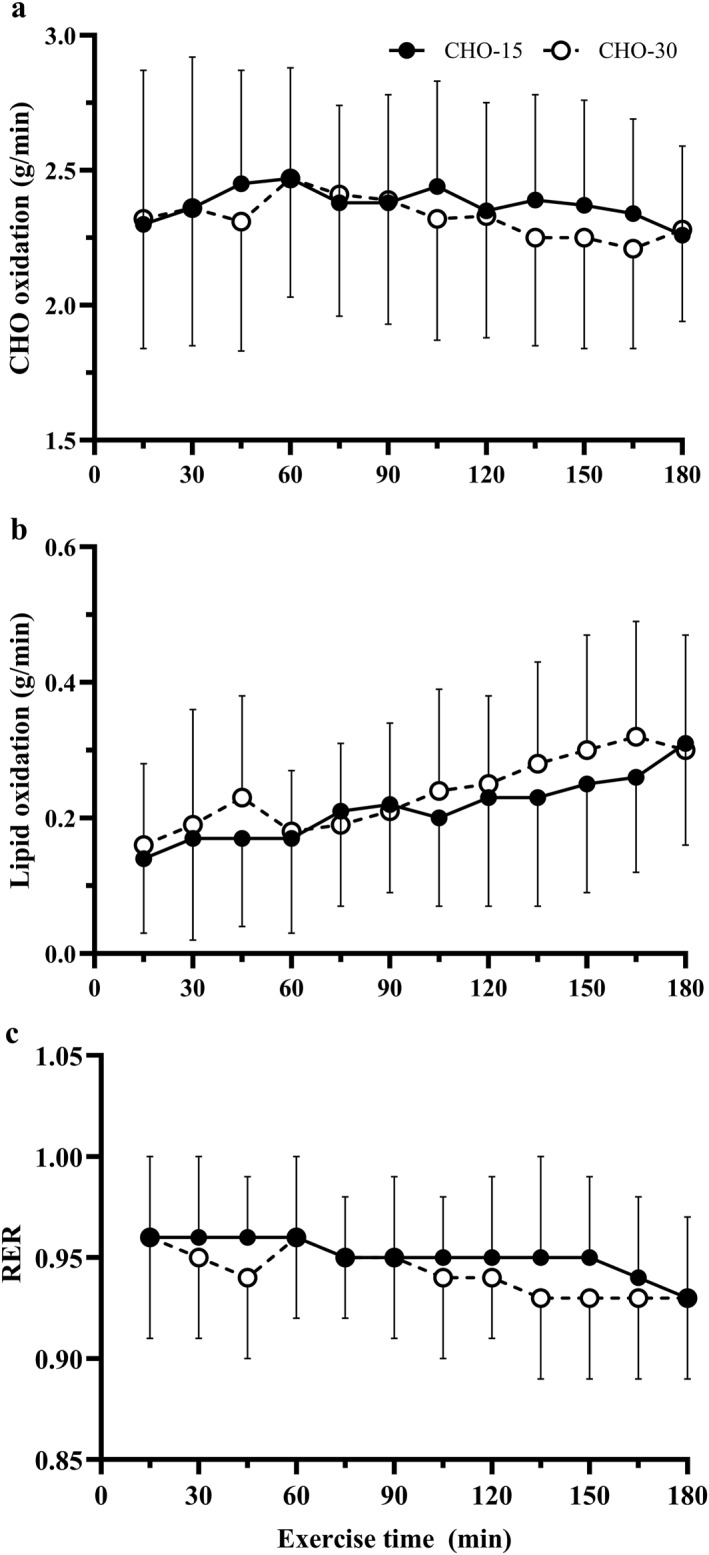
Carbohydrate oxidation (a), fat oxidation (b) and respiratory exchange ratio (c) throughout 180 min steady state cycling with high carbohydrate availability through different carbohydrate ingestion pattern. ^a^ Significantly different from CHO‐15.

RER showed no main effect for condition (*p* = 0.16, Figure 3c) or interaction (*p* = 0.18) and decreased steadily throughout the exercise period for both conditions (−0.03 from 15 to 180 min, time effect *p* < 0.001 and *η* ^2^_*p*_ = 0.30). Mean whole body CHO oxidation (2.38 ± 0.37 and 2.31 ± 0.39 g/min for CHO‐15 and CHO‐30, respectively) was similar in both conditions (*p* = 0.25 and *η* ^2^_*p*_ = 0.07; Figure 3), with no main effect for time or interaction (*p* = 0.09, *η* ^2^_*p*_ = 0.10 and *p* = 0.11, *η* ^2^_*p*_ = 0.09 respectively; Figure 3). Fat oxidation was not significantly affected by condition (*p* = 0.10 and *η* ^2^_*p*_ = 0.14) and increased ∼2‐fold by 180 min of exercise (*p* < 0.001 and *η* ^2^_*p*_ = 0.44), which reached significance compared to the first time point at 120 min and thereafter (*p* < 0.05). There was no significant interaction effect for fat oxidation (*p* = 0.09).

Exercise energy expenditure increased over time (*p* < 0.001) reaching significance compared to the first time point following 60 min of exercise (*p* = 0.01). There was no main effect for condition (*p* = 0.92) or significant interaction (*p* = 0.51) as exercise energy expenditure was comparable for both conditions (Table 2). Mean contribution of CHO to total exercise energy expenditure was 84% ± 9% and 82% ± 10% for CHO‐15 versus CHO‐30, respectively, with the remaining 16% and 18% being accounted for by fat utilisation (*p* = 0.07). Throughout exercise time, % CHO decreased in line with the % increase in fat utilisation (*p* < 0.001) reaching statistical significance at 165 min of exercise. There was no time‐condition interaction (*p* = 0.08).

**FIGURE 4:**
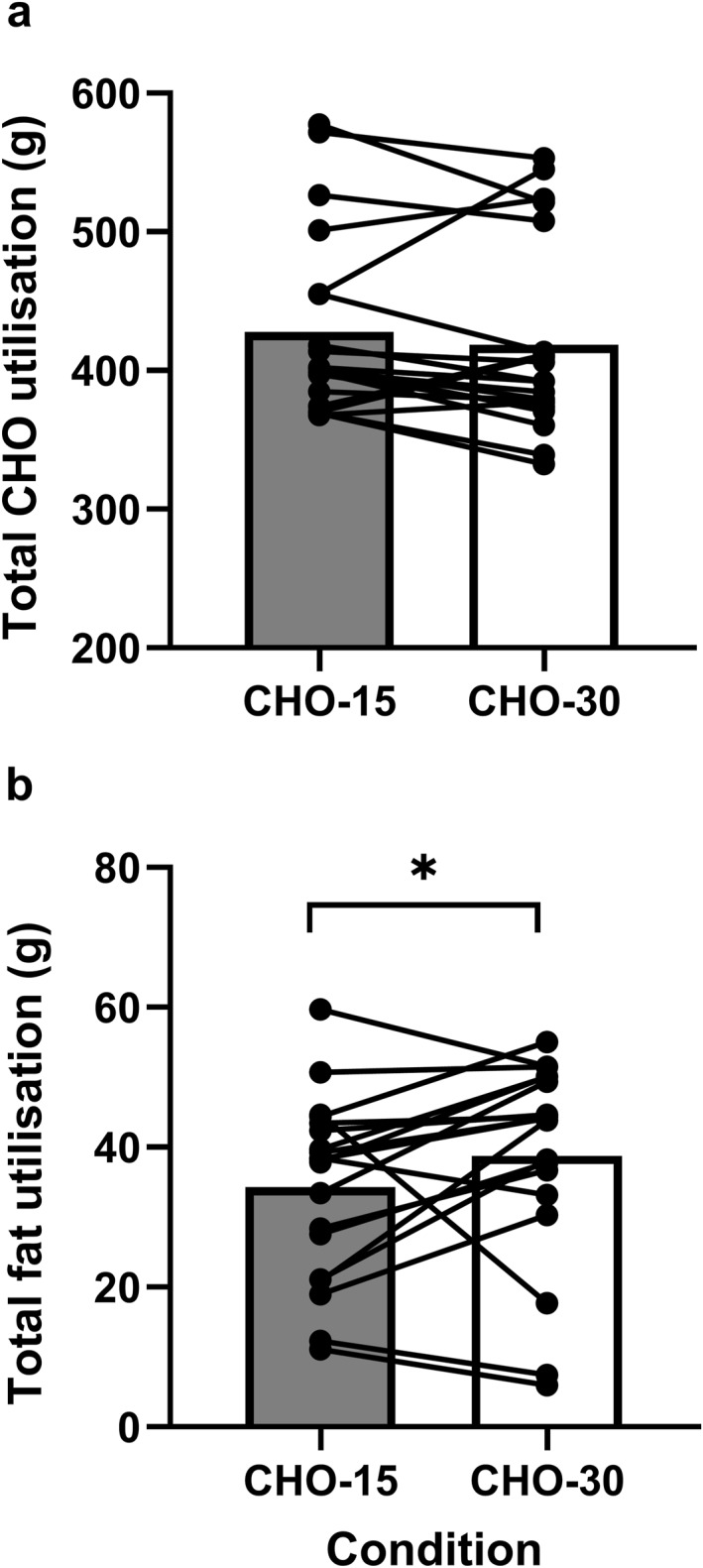
Total carbohydrate (a) and fat utilisation (b) with individual data points, during 180 min steady state cycling with high carbohydrate availability through different carbohydrate ingestion patterns. * Indicates significant difference between conditions (*p* = 0.04).

There was no significant difference between total CHO utilised in CHO‐15 versus CHO‐30 (*p* = 0.13; Figure 4). However, there was a moderate effect size (ES = 0.48), as median CHO utilisation was 401.8 and 392.3 g for CHO‐15 and CHO‐30, respectively. There was a significant difference between CHO‐15 and CHO‐30 in total fat utilised (*p* = 0.04 and ES = 0.39; Figure 4) as median fat utilisation was 34.3 and 38.7 g for CHO‐15 and CHO‐30, respectively.

### 4.3 Subjective Responses

Mean subjective scores for GI symptoms did not differ between conditions (Tables 3 and 4), except for the urge to defecate; however, this was not considered meaningful as the highest reported score was ≤ 3. There was no time main effect for symptoms of stomach fullness, abdominal cramps or urge to defecate. Nausea and gas/flatulence showed a trend, as scores increased with exercise time and CHO ingestion. Reflux showed a significant main effect for time, with increased symptoms during the final 1 h of exercise; however, no individual participant scored > 4 for nausea, gas/flatulence or reflux across any time point. There was no interaction effect across any GI discomfort symptoms. No participant scored > 4 for any variable in either condition, except for stomach fullness, which saw a trend, due to one participant who reported a peak score of 7 for CHO‐15 at 165 and 180 min.

**TABLE 3:** GI symptom scores during 180 min steady state exercise with high carbohydrate availability through consumption of either 22.5 g every 15 min (CHO‐15) or 45 g every 30 min (CHO‐30).

Condition	Exercise time (min)	Nausea (0–10)	Reflux (0–10)	Stomach fullness (0–10)	Cramps (0–10)	Flatulence/gas (0–10)	Urge to defecate (0–10)
CHO‐15	15	0 ± 0	0 ± 0	1 ± 2	0 ± 0	0 ± 0	0 ± 0
	30	0 ± 0	0 ± 0	1 ± 2	0 ± 0	0 ± 0	0 ± 0
	45	0 ± 0	0 ± 0	1 ± 2	0 ± 0	0 ± 0	0 ± 0
	60	0 ± 0	0 ± 0	1 ± 2	0 ± 0	0 ± 0	0 ± 1
	75	0 ± 0	0 ± 0	1 ± 2	0 ± 0	0 ± 0	0 ± 1
	90	0 ± 1	0 ± 1	1 ± 2	0 ± 1	0 ± 0	0 ± 1
	105	0 ± 0	0 ± 1	1 ± 2	0 ± 0	0 ± 0	0 ± 1
	120	0 ± 1	0 ± 1	1 ± 2	0 ± 0	0 ± 1	0 ± 1
	135	0 ± 0	1 ± 1	1 ± 2	0 ± 0	0 ± 1	0 ± 0
	150	0 ± 1	1 ± 1	1 ± 2	0 ± 0	0 ± 1	0 ± 0
	165	0 ± 1	1 ± 1	1 ± 2	0 ± 0	0 ± 1	0 ± 0
	180	0 ± 1	0 ± 1	1 ± 2	0 ± 0	0 ± 1	0 ± 1
CHO‐30	15	0 ± 0	0 ± 0	1 ± 2	0 ± 0	0 ± 0	0 ± 0
	30	0 ± 0	0 ± 0	1 ± 2	0 ± 1	0 ± 0	0 ± 0
	45	0 ± 0	0 ± 0	1 ± 2	0 ± 0	0 ± 0	0 ± 1
	60	0 ± 0	0 ± 0	1 ± 2	0 ± 0	0 ± 0	0 ± 0
	75	0 ± 0	0 ± 0	1 ± 2	0 ± 0	0 ± 0	0 ± 0
	90	0 ± 0	0 ± 0	1 ± 2	0 ± 0	0 ± 0	0 ± 0
	105	0 ± 0	0 ± 1	1 ± 2	0 ± 0	0 ± 0	0 ± 0
	120	0 ± 0	0 ± 0	1 ± 2	0 ± 0	0 ± 1	0 ± 0
	135	0 ± 1	0 ± 1	1 ± 2	0 ± 1	0 ± 1	0 ± 0
	150	0 ± 1	0 ± 1	1 ± 2	0 ± 0	0 ± 1	0 ± 0
	165	1 ± 1	1 ± 1	1 ± 2	0 ± 0	0 ± 1	0 ± 0
	180	0 ± 1	1 ± 1	1 ± 2	0 ± 0	1 ± 1	0 ± 0

**TABLE 4:** Two‐way ANOVA test statistics for GI symptom scores during 180 min steady state exercise with high carbohydrate availability through consumption of either 22.5 g every 15 min (CHO‐15) or 45 g every 30 min (CHO‐30).

GI symptom	Condition effect	Time effect	Interaction effect
Nausea	*p* = 0.56	*p* = 0.07	*p* = 0.23
Reflux	*p* = 0.24	***p* = 0.04**	*p* = 0.19
Stomach fullness	*p* = 0.36	*p* = 0.76	*p* = 0.07
Cramps	*p* = 0.67	*p* = 0.44	*p* = 0.37
Flatulence/Gas	*p* = 0.95	*p* = 0.06	*p* = 0.57
Urge to defecate	***p* = 0.05**	*p* = 0.30	*p* = 0.18

*Note:* Bold values indicate statistical significance.

Perceptions of sweetness saw a significant interaction (*p* = 0.04) as scores increased with exercise time (*p* = 0.06), the increase was greater in CHO‐30 versus CHO‐15 (+0.9 and +0.4, respectively). Mean perceptions of sweetness scores were 5.6 ± 1.9 and 6.1 ± 1.6 for CHO‐15 and CHO‐30, respectively (no main effect for condition: *p* = 0.14). Mean desire to consume CHO were 2.0 ± 2.5 and 2.1 ± 2.0 (no main effect for condition: *p* = 0.69 and interaction effect: *p* = 0.62), which was consistent over time (time effect: *p* = 0.35).

### 4.4 Exercise Capacity

There was no difference (*p* = 0.79 and ES = 0.08) in exercise capacity between CHO‐15 and CHO‐30 as median capacity time was 9 and 8 min 25 s, respectively.

## 5 Discussion

The primary aim of the study was to investigate the effects of different CHO ingestion patterns (22.5 g every 15 min vs. 45 g every 30 min) on physiological responses to exercise, whole‐body substrate oxidation and GI symptoms during endurance cycling with high CHO availability. As hypothesised, there was no meaningful difference in physiological responses to exercise or whole‐body substrate oxidation between conditions. Furthermore, both strategies were well tolerated, with minimal GI symptoms reported. Overall, current study data suggest the larger less frequent CHO feeding frequency used in the current study is a feasible, practical nutritional strategy for endurance cycling.

Physiological responses to exercise were not significantly different between conditions with no significant interactions for heart rate, RPE, absolute V̇O_2_ or blood lactate, respectively (Table 2). The less frequent higher CHO dose (45 g CHO every 30 min) showed no exacerbated exercise induced stress, perceived exertion or oxygen demand compared to the frequent lower dose CHO ingestion pattern (22.5 g every 15–20 min) more commonly utilised during competition and laboratory‐based studies (E. F. Coyle et al. 1986; Jentjens et al. 2004; Fell et al. 2021; Hearris et al. 2022), thus providing an alternative feeding strategy. Despite feeding being less disruptive compared to sports, such as running or skiing, where athletes are required to slow down to ingest CHO, endurance cycling under race conditions with regular feedings every 15–20 min (as required to achieve the recommended CHO intake of 90–120 g/h) can be tedious or impractical. A larger less frequent dose or perhaps a mixed flexible approach throughout competition could better suit athletes.

As hypothesised, whole body CHO oxidation rates were similar in both conditions (Figure 3). These values are comparable to Mears et al. (2020), where ingestion of a sports drink (60 g/h) at a feeding frequency of every 5 or 20 min resulted in whole body CHO oxidation rates of 2.23 ± 0.45 and 2.15 ± 0.47 g/min, respectively. Mears et al. (2020) reported 23% higher exogenous CHO oxidation in the larger less frequent ingestion pattern (200 mL every 20 min), despite previous literature suggesting a similar pattern (increased oxidation during initial 75–90 min, followed by plateau) and peak rate of exogenous CHO oxidation during exercise when ingesting a 100 g glucose bolus (Krzentowski et al. 1984; Guezennec et al. 1989) compared to more frequent feedings every 20 min (Massicotte et al. 1989, 1990, 1994). Unfortunately, it cannot be confirmed which response would occur in the current study, as exogenous CHO was not specifically measured. However, Mears et al. (2020) attributed differences in exogenous CHO oxidation to gastric emptying, where the larger bolus provided greater total volume of fluid per feeding, increasing gastric pressure and emptying, which allowed earlier absorption and subsequent utilisation of CHO (N. J. Rehrer et al. 1992; Noakes et al. 1991; Costill and Saltin 1974). Current study conditions were not perfectly matched for total volume of fluid ingested (840 and 720 mL/h for CHO‐15 and CHO‐30, respectively) as both CHO gels provided 60 mL of fluid per feeding as well as 150 mL of water every 15 min, suggesting that gastric emptying, and perhaps exogenous CHO oxidation, could have differed between conditions. However, differences in absolute CHO content of ingested solutions have previously been shown to decrease gastric emptying (N. Rehrer et al. 1990), but increase exogenous CHO oxidation (N. J. Rehrer et al. 1992), as a reduction in gastric emptying does not necessarily mean a reduction in CHO absorption (Noakes et al. 1991). Therefore, it is possible, that both these regulatory factors counteracted one another to maintain a similar exogenous CHO oxidation rate between current study conditions. The use of stable isotope tracers in future cycling studies is required to confirm the exact effects of different CHO ingestion patterns, as utilised in the current study, on endogenous and exogenous oxidation rates.

In agreement with Mears et al. (2020), fat oxidation (g/min) was not significantly different between CHO ingestion patterns utilised in the current study. In contrast, Stocks et al. (2016) showed high CHO intake over low frequency of ingestion during cross‐country skiing to decrease fat oxidation, which elicited a greater reliance on CHO from endogenous glycogen stores. Such a response has potential to negatively impact exercise performance due to earlier onset of fatigue (E. Coyle 1992; Stellingwerff and Cox 2014). There may be a threshold whereby a large bolus, too infrequently, will lead to metabolic perturbations that are unfavourable for endurance performance due to highly elevated insulin concentrations during exercise, which blunt lipid mobilisation, decreasing fat oxidation (A. E. Jeukendrup et al. 1999). Nonetheless, in the current study, substrate oxidation data (Figure 3) suggest the lower frequency ingestion strategy utilised (45 g CHO every 30 min) was below this threshold, supporting its use as a practical feeding strategy. In fact, total fat oxidation was greater in CHO‐30 compared to CHO‐15 (*p* = 0.04 and ES = 0.39), with a large effect size expressed over exercise time (*η* ^2^_*p*_ = 0.14). Whether this corresponded to a sparring of endogenous glycogen stores through increased fat utilisation or would be a meaningful difference in practice remains unclear.

Blood glucose was comparable between conditions, with euglycemia maintained throughout the 180 min of exercise in both conditions (Figure 2a). However, there was an initial decrease observed following 15 min of cycling, likely due to the high CHO breakfast ∼60 min pre‐exercise. This is a well‐established phenomenon, where the combined effects of post‐prandial insulin‐mediated glucose uptake into the muscle and blunted hepatic glucose production, as well as further augmented glucose uptake via exercise induced activation of calcium dependent GLUT 4 transporters, results in a mismatch between glucose uptake into the muscle and rate of appearance in the blood (Ahlborg and Felig 1977; Costill et al. 1977; E. F. Coyle et al. 1997; A. E. Jeukendrup and Killer 2010). Following 30 min of exercise, glucose increased for both conditions, with the greater increase in CHO‐15 caused by exogenous CHO gel ingestion. Any early increased reliance on endogenous stores (pre‐exogenous CHO provision) was transient and only occurred during the initial 30 min of exercise, likely minimally impacting the rate of glycogen depletion or the onset of fatigue (Gleeson et al. 1986; Sherman et al. 1991), as supported by the current study RPE and exercise capacity data where responses between conditions were matched. It should be noted, earlier provision of exogenous CHO during exercise (as in CHO‐15) would be more beneficial in restoring blood glucose concentrations and avoid potential negative symptoms of rebound hypoglycaemia (blood glucose < 3.5 mmol/L). However, appropriate timing of the pre‐exercise meal (at least 1–4 h pre‐exercise) or consumption of CHO immediately pre‐exercise would minimise this risk (A. E. Jeukendrup and Killer 2010).

Mean GI discomfort symptoms did not differ between conditions for any symptom other than the urge to defecate, where no participants recorded a score > 3, confirming that any discomfort was minimal (Wilson 2017). This is in line with Mears et al. (2020), where sports drink ingestion every 5 min, or 20 min (60 g/h in both conditions), showed minimal discomfort symptoms. In contrast, in Stocks et al. (2016) well‐trained cross‐country skiers reported higher GI discomfort symptoms when consuming higher quantities of CHO at less frequent intervals, likely a result of the specific ingestion pattern utilised, where participants were provided with two large boluses (686 ± 83 mL) of a 24% CHO solution 5 min prior to, and during a high intensity performance test. Both the higher CHO dose and exercise intensity (potentially through restricted GI blood flow [Brouns and Beckers 1993]) contributed to differences compared to the current study, where participants consumed much smaller more tolerable quantities of CHO (22.5 and 45 g as 60 mL CHO gels every 15 and 30 min respectively) during a light–moderate intensity steady state cycling bout (∼60% V̇O_2max_). Again, this highlights a threshold where CHO ingestion patterns (a dose too large, too infrequently) may negatively impact gut comfort and result in a suboptimal physiological condition for endurance cycling performance. However, other confounding methodological differences (participant training status, type of CHO consumed, CHO form [gel vs. drink] and exercise mode) makes identifying exactly where this threshold occurs impossible currently in endurance cycling. That being said, both strategies utilised in the current study were effective with minimal impact of GI discomfort.

Exercise capacity at 150% of LT1 following 180 min steady state cycling did not differ between CHO‐15 and CHO‐30, which was expected, as participants in both conditions consumed the same absolute quantity of CHO during exercise (90 g/h), and an almost identical study protocol from our lab (prescribed nutritional intake and exercise [type, intensity and duration]) showed similar results in an endurance trained population whilst feeding 120 g/h (Hearris et al. 2022). The variable nature of such tests due to psychological factors (motivation/boredom) may have limited the ability to determine small differences between conditions (A. Jeukendrup et al. 1996). However, all participants seemingly provided a maximal effort, which, combined with the large study sample size, should have minimised this effect.

To the authors knowledge, this is the first‐time participant perceptions of sweetness and desire to consume CHO during endurance exercise was measured. Perceptions of sweetness increased in both conditions; however, the increase was greater during CHO‐30 as participants found the larger CHO gels to be sweeter, presumably due to the greater absolute quantity of glucose (30 g) and fructose (15 g) per feeding. Of note, mean values between conditions were similar, both being close to the optimal value of 5, suggesting the strategies were well tolerated. However, participant desire to consume CHO throughout exercise was consistently low across both conditions. Despite near optimal sweetness and minimal GI discomfort symptoms, participants had very little desire to consume CHO. This may be attributed to a lack of understanding of the importance of CHO for endurance exercise, or simply a lack of experience consuming nutritional products during exercise, as a high rate of CHO ingestion during exercise (90 g/h) was a new experience for all but two study participants.

One limitation of the study was the participant training status, despite being healthy and recreationally active, participants were not endurance trained and were unfamiliar with consuming high CHO quantities during exercise, which may have impacted study results. Therefore, care should be taken not to generalise findings to endurance trained individuals, without considering the metabolic differences between populations. Another limitation was poor blinding of participants to exercise capacity time. Despite not being directly told by researchers, an estimate was visible through total exercise time on display, which could have impacted participant motivation, hence the little emphasis placed on these data throughout.

## 6 Conclusions

The use of a larger CHO dose at less frequent feeding intervals in endurance cycling, as utilised in the current study (45 g every 30 min), is a feasible and perhaps more practical nutritional strategy, with no meaningful negative impact on physiological responses to exercise, whole‐body substrate oxidation or GI discomfort compared to the more commonly used feeding frequency (22.5 g every 15 min). However, different CHO ingestion patterns are an area with little research focus, particularly in cycling models. Further work is needed, in well‐trained cyclists, with the use of stable isotope tracers to confirm the impact of different strategies on the contribution of exogenous and endogenous CHO to overall exercise energy expenditure, and ultimately, performance.

## Conflicts of Interest

The authors declare no conflicts of interest.
